# Selective antagonism of cJun for cancer therapy

**DOI:** 10.1186/s13046-020-01686-9

**Published:** 2020-09-11

**Authors:** Andrew Brennan, James T. Leech, Neil M. Kad, Jody M. Mason

**Affiliations:** 1 Department of Biology & Biochemistry, University of Bath, Claverton Down, Bath, BA2 7AY UK; 2 School of Biosciences, University of Kent, Canterbury, CT2 7NH UK

**Keywords:** c-Jun, Activator Protein-1, transcriptional regulator, basic leucine zipper, cancer, peptides, protein-protein interaction

## Abstract

The activator protein-1 (AP-1) family of transcription factors modulate a diverse range of cellular signalling pathways into outputs which can be oncogenic or anti-oncogenic. The transcription of relevant genes is controlled by the cellular context, and in particular by the dimeric composition of AP-1. Here, we describe the evidence linking cJun in particular to a range of cancers. This includes correlative studies of protein levels in patient tumour samples and mechanistic understanding of the role of cJun in cancer cell models. This develops an understanding of cJun as a focal point of cancer-altered signalling which has the potential for therapeutic antagonism. Significant work has produced a range of small molecules and peptides which have been summarised here and categorised according to the binding surface they target within the cJun-DNA complex. We highlight the importance of selectively targeting a single AP-1 family member to antagonise known oncogenic function and avoid antagonism of anti-oncogenic function.

## Background

Activator protein-1 (AP-1) designates a family of oncogenic transcription factors (TFs) that are integral components located at the end of a number of key signalling networks, controlling vital cellular processes such as differentiation, migration, proliferation and apoptosis [1–6]. AP-1 functions as homo- or hetero-dimeric combinations of proteins in the Fos and Jun sub-families (a broader definition of AP-1 includes ATF and MAF sub-families) [7, 8]. As a dimer, AP-1 binds to cognate DNA sites within gene promotor elements to influence the expression of a range of target genes that include cyclin D1, FasL, SDF1, TNFα, proliferin and CD44 [9–11]. This review will focus on cJun, an AP-1 family member which is found to be upregulated or overexpressed in a large number of cancers (for a list of cancers associated with specific AP-1 members, see Table 1) [47–50]. cJun has since become a major focus for drug discovery, and its terminal activity within a number of pathways makes for a compelling target to ablate oncogenic signals that occur at any signalling level. This review will describe the role of AP-1 in general and how cJun specifically is dysregulated in various cancers. We describe antagonists from the literature, categorised according to the interaction surface within the cJun-DNA complex they target, as potential therapeutics against cJun dysregulation.

**Table 1:** Examples of specific cancer types linked to dysregulated activity of AP-1 family members. In brackets we have indicated whether evidence points to up- or down-regulation of the AP-1 family member

AP-1 family member	Type of cancer	Up/down-regulation	References
cJun	Breast cancer	Upregulation	[12, 13]
	Colorectal cancer	Upregulation	[14]
	Fibrosarcoma	Upregulation	[15]
	Glioma	Upregulation	[16]
	Hodgkin lymphoma	Upregulation	[17]
	Lung cancer	Upregulation	[18]
	Myeloid leukaemia	Upregulation	[19]
	Urothelial carcinoma of the bladder	Upregulation	[20]
JunB	Breast cancer	Upregulation	[21]
	Cervical cancer	Upregulation	[22]
	Colon cancer	Upregulation	[23]
	Fibrosarcoma	Upregulation	[15]
	Head and neck squamous cell carcinoma	Upregulation	[24]
	Hodgkin lymphoma	Upregulation	[17]
	Prostate cancer	Downregulation	[25]
JunD	Cervical cancer	Upregulation	[22]
	Prostate cancer	Upregulation	[26, 27]
cFos	Breast cancer	Upregulation	[28]
	Cervical cancer	Upregulation	[22]
	Colon cancer	Upregulation	[23]
	Gastric cancer	Downregulation	[29]
	Head and neck squamous cell carcinoma	Upregulation	[30]
	Ovarian cancer	Downregulation	[31]
	Pancreatic cancer	Upregulation	[32]
	Skin cancer	Upregulation	[33]
	Tongue cancer	Upregulation	[34]
	Urothelial carcinoma of the bladder	Upregulation	[20]
FosB	Breast cancer	Downregulation	[35]
	Colon cancer	Downregulation	[23]
	Gastric cancer	Downregulation	[36]
	Non-small cell lung cancer	Downregulation	[37]
	Ovarian cancer	Upregulation	[38]
	Pancreatic cancer	Downregulation	[39]
Fra1	Breast cancer	Upregulation	[40]
	Cervical cancer	Downregulation	[22]
	Colon cancer	Upregulation	[41]
	Liver cancer	Upregulation	[42]
	Lung cancer	Upregulation	[43]
	Skin and hand and neck squamous cell carcinoma	Upregulation	[44]
Fra2	Breast cancer	Upregulation	[45]
	Non-small cell lung cancer	Upregulation	[46]
	Tongue cancer	Upregulation	[34]

## AP-1 Structure and Function

**Fig. 1:**
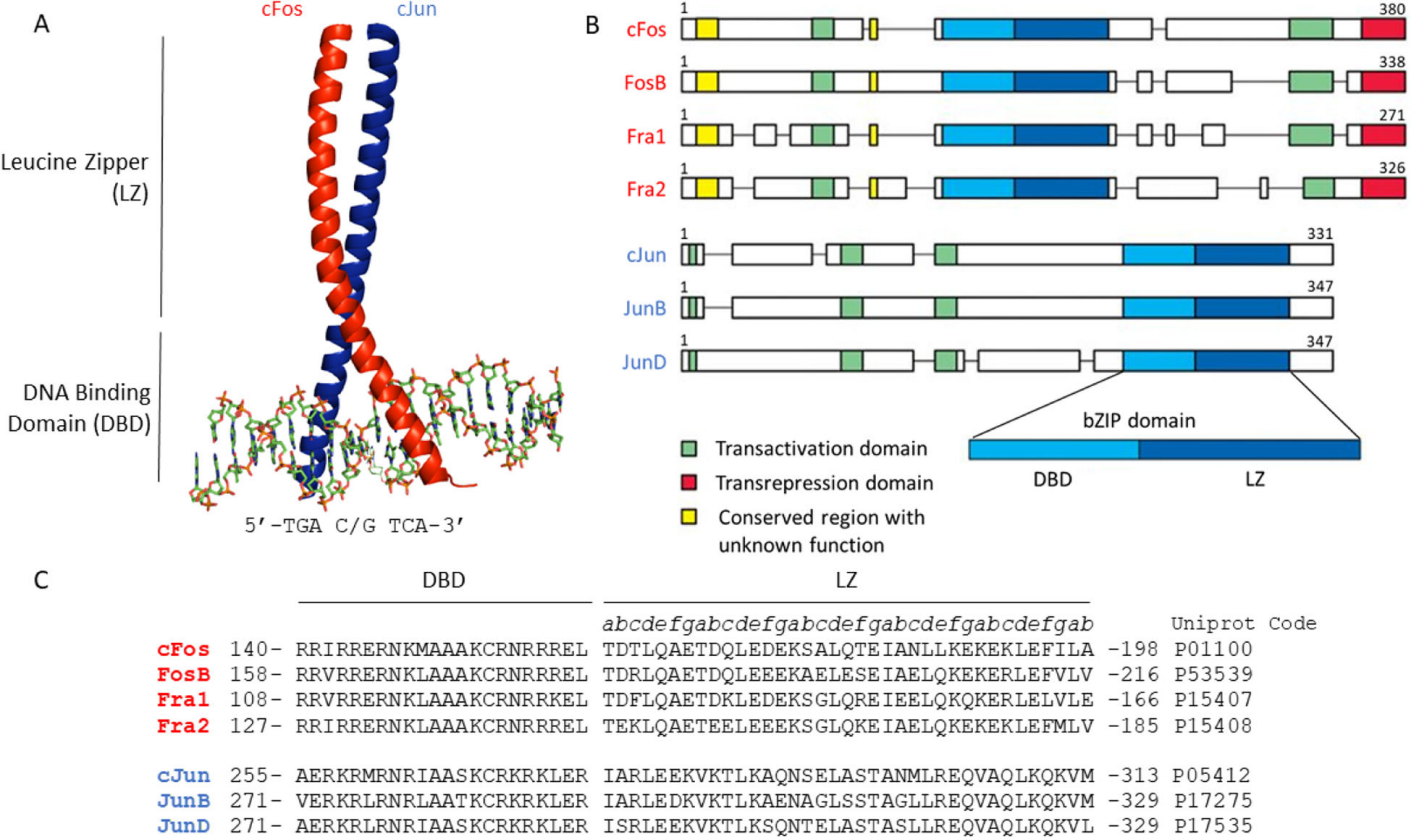
Sequence and structure of AP-1 proteins. **a** Crystal structure of the cFos-cJun heterodimer binding to DNA (PDB code: 1FOS); **b** Schematic illustrating the domain structures of AP-1 proteins, adapted from Ref [51]; **c** Sequence alignment of the bZIP domains from selected AP-1 proteins

AP-1 proteins bind to DNA via their basic leucine-zipper (bZIP) domain (Fig 1a); comprised of an N-terminal DNA binding domain (DBD) and C-terminal leucine zipper (LZ). The LZ is the site of AP-1 dimerisation where an intermolecular interaction is facilitated by the formation of an α-helical coiled coil. This incorporates hydrophobic packing of **i**, **i+7** repeating aliphatic hydrophobic residues (***a*** position of the heptad repeat), and leucine residues (**d** position) which are flanked by polar and charged residues (**e** and **g** positions). The DBD forms an N-terminal extension of the α-helices from each protein to grip the DNA in a manner comparable to forceps. This inserts into the DNA major groove where basic sidechains interact favourably with the DNA phosphate groups, while key residues form specific hydrogen bonding contacts with bases within the recognition sequence [52, 53].

AP-1 proteins bind to a pseudo-palindromic DNA sequence (5’-TGA C/G TCA-3’) known as the 12-O-tetradecanoylphorbol-13-acetate (TPA) response element (TRE) [54–56]. The DBD of each AP-1 protein within a given dimer binds to a separate half site (5’-TGA-3’) on the sense/antisense strands, separated by a C/G spacer base. The cFos-cJun heterodimer has shown binding promiscuity to single nucleotide variants (SNVs) of the TRE sequence with up to a 40-fold variation in affinity [53]. SNVs of the TRE site, present throughout the human genome, likely provide a layer of modulation to the ability of AP-1 dimers to alter the transcription of genes where they occur. AP-1 dimers may also bind to the cAMP response element (CRE, 5’-TGA CG TCA-3’) with a relevant though weaker affinity [57]. Crystal structures of the cJun homodimer interacting with both TRE and CRE site DNA are available (PDB codes: 1H7H for TRE, 1JNM for CRE). The binding affinities for these related DNA sites is controlled by AP-1 dimer composition, which in some cases leads to changes in binding site preference (particularly within the broader definition of AP-1 which includes Jun/Fos-ATF/MAF heterodimers) [58, 59]. In totality, this produces a regulatory system where the expression level of individual AP-1 proteins, and their subsequent nuclear transport, control the amounts of each AP-1 dimer available to bind [60–62]. The sequence of a potential DNA binding site (TRE, CRE or SNVs thereof) will then define the affinity for each dimer present. This allows global alterations in AP-1 expression, and therefore dimer composition, induced by signalling events to produce a fine-tuned effect at a specific site of transcription.

All AP-1 family members have transactivation (TA) domains but only the Fos sub-family have been shown to have transrepression (TR) domains; found at their C-terminus (Fig 1b) [63–67]. Generally, these domains have been identified by mutation/deletion of residues/regions though little further mechanistic study has been carried out. The transactivational activities of cJun, cFos and FosB are considered strong whereas activities for JunB, JunD, Fra-1 and Fra-2 are considered weak or repressive [50, 68]. This activity will be modulated by both the bZIP-DNA binding affinity and the activity of the domains themselves, which have some sequence diversity and therefore presumably differences in activity. As with DNA site specificity, the transactivation activity is modulated by AP-1 dimer composition.

## AP-1 Activity

The activity of AP-1 is controlled by the cellular context within which it is operating. This cellular context can be thought of in terms of AP-1 protein expression levels defining the prominent dimer compositions; the post translational modification of AP-1 proteins which can enhance or diminish activity; and the genetic organisation within a given cell as defined by epigenetics. The importance of cellular context is highlighted by the pro-apoptotic activity of cJun in neurons, in contrast with the anti-apoptotic activity of cJun in hepatocytes [68]. The various roles of AP-1 components have been the subject of significant study using gene knockout (KO) and transgenic mice, as discussed thoroughly in a review by Wolfram *et al* [50]. They describe the embryonic lethality of cJun, JunB and Fra-1 (showing they are indispensable) but KO of any AP-1 causes some detrimental effect such as osteopetrosis (cFos KO) or male sterility (JunD KO). AP-1 KO and transgenic mice were phenotypically diverse implying clear differences in the function of these proteins. In addition to observing the effect of AP-1 family protein KO, specific genes which are under AP-1 transcriptional control have been elucidated. AP-1 cellular control is mediated through activity on genes including cell-cycle regulators such as cyclin D1, cyclin A, apoptotic proteins such as FasL and TNF-α, chemokines such as SDF1 and many others [9–11, 69, 70].

**Fig. 2:**
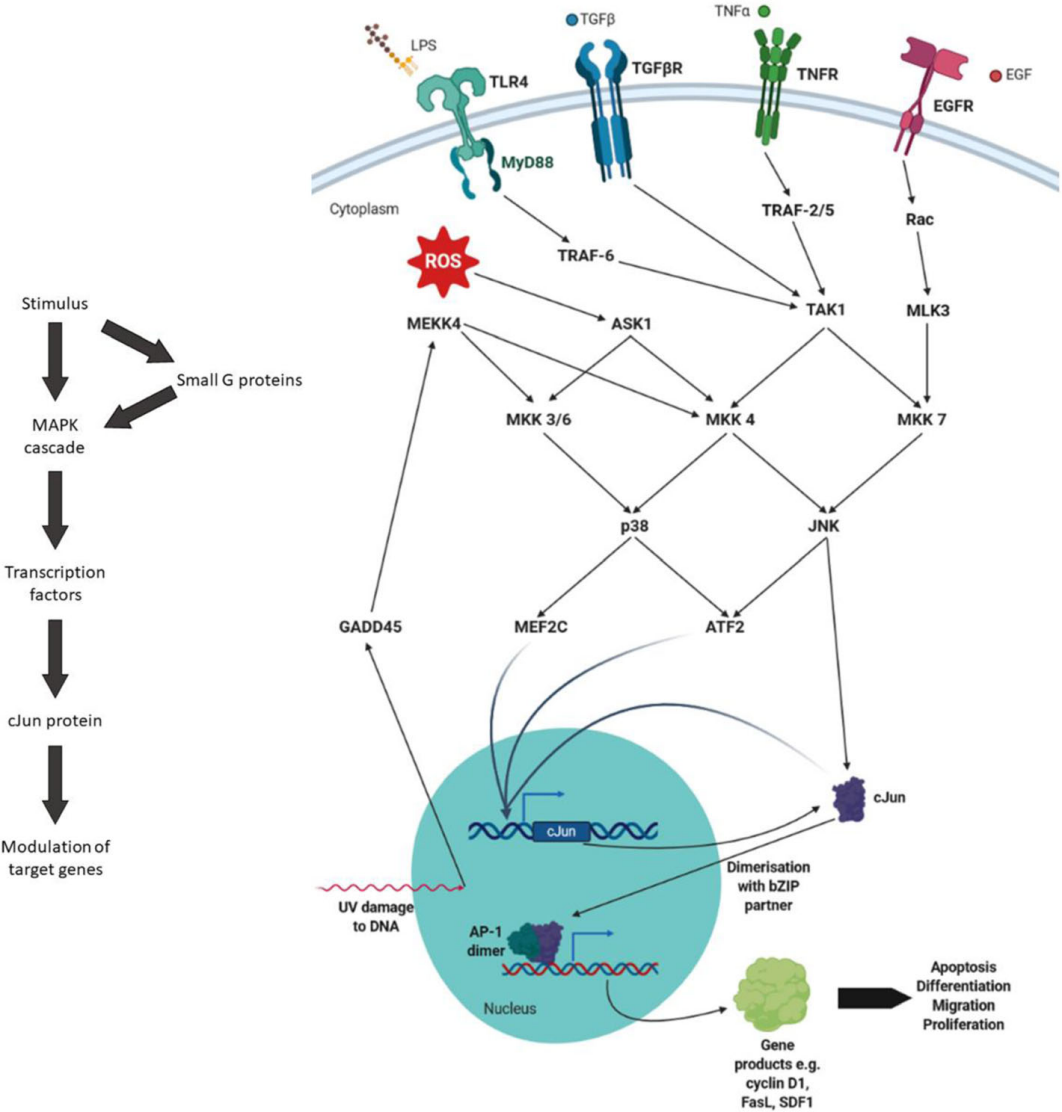
Overview of selected cJun signalling pathways. In general, a stimulus is passed through a MAPK cascade (sometimes via a small G protein) to alter the activity of transcription factors which act upon the cJun gene; this changes expression of target genes by the formation of an AP-1 dimer which binds to TRE DNA. Exemplary pathways have been shown which are indicative of the general signalling through which a stimulus leads to cJun mediated cellular changes. The pathways are therefore not necessarily complete as well as some of these activations occurring indirectly. Created with Biorender.com

AP-1 proteins respond to numerous environmental and cellular stimuli including cytokines such as TNFα, hormones and neurotransmitters such as growth hormone, growth factors such as EGF, bacterial lipopolysaccharide (LPS), UV damage to DNA and reactive oxygen species [1–5, 71–78]. Each stimulus produces a signalling cascade which can alter the activity of AP-1 by changing transcription or by direct activation. The signal is passed through a mitogen-activated protein kinase (MAPK) cascade (sometimes initiated by a small G protein such as RAS or Rac) which terminally acts upon transcription factors. cJun N-terminal kinase (JNK) and p38 have both been shown to act in this manner by their phosphorylation of transcription factors including myocyte-specific enhancer factor 2C (MEF2C), activating transcription factor 2 (ATF2) and cJun [79, 80]. It is known that MEF2C, ATF2 and cJun itself are able to influence cJun expression [81–84]. JNK increases cJun transactivation activity through phosphorylation of Ser63 and Ser73 within the TA domain [79]. JNK phosphorylation has also been shown to decrease ubiquitination-dependent proteasomal degradation of cJun which effectively increases cJun activity by virtue of increased cJun levels [85]. The array of interconnected components within the AP-1 signalling system allows the transduction of multiple pathways; balancing signals to define transcription and allow AP-1 to assert its role in cellular processes. Some cJun pathways that generalise aspects of AP-1 signalling are shown in Fig 2.

## AP-1 as an Oncoprotein

Although AP-1 dysfunction has been implicated in other pathological conditions including asthma and rheumatoid arthritis, cancer is the most prominently studied and is the focus here [47–51, 86, 87]. Some hallmarks of cancerous cells which can be linked with AP-1 dysfunction are growth signal autonomy, angiogenesis, lack of apoptosis and uncontrolled cell migration. The functional role of AP-1 proteins has been shown to vary, resulting in a mixture of oncogenic and anti-oncogenic effects, depending upon cellular context and dimer composition [48]. For example, a cJun homodimer tends to have oncogenic activity however a cJun-JunB heterodimer can repress that function [88]. We may look to the specific case of breast cancer where cJun has been shown to be upregulated whereas FosB is downregulated [12, 13, 35]. In this context, therefore, AP-1 family members must be considered and treated individually. We focus on the role of cJun which has generally been shown to have an oncogenic effect. Early studies on the role of cJun in cancer showed sequence homology with known viral oncoprotein vJun, and overexpression of cJun was capable of malignantly transforming rat embryonic cells towards a cancer phenotype [67, 89, 90]. Although this alone did not imply cJun overexpression as an *in vivo* cause of human cancer, it was a strong indicator of the involvement of cJun and led to an intense period of further research.

Genetic studies can illuminate the target genes through which cJun exerts influence to produce hallmark cancer cell behaviours. cJun has been shown to regulate a range of genes involved in tumour development including cyclin D1 (upregulated to stimulate proliferation), Fas (downregulated to inhibit apoptosis), proliferin (upregulated to stimulate angiogenesis) and CD 44 (upregulated to stimulate invasiveness) [48, 91–95]. However, the picture is not simplistic as cJun may also contribute anticancer effects, for example it upregulates BCL-2 interacting mediator of cell death (BIM) which stimulates apoptosis [96]. cJun appears to be best described as an oncoprotein but there is balance in its effects; tempered by cellular context.

### cJun in Cancer

Evidence for the role of cJun in cancer has been developed by quantifying the amount of cJun in various primary cancer tissue samples to create a correlative link. One such study with lung cancer patients demonstrated cJun overexpression in 31% of the tumour samples tested [18]. The involvement of cJun was illustrated via immunohistochemistry which could not detect cJun in normal conducting airway and alveolar epithelial cells, but it was found in histologically atypical areas. Another example of this difference was observed in colorectal adenocarcinoma tumour samples, where cJun was found to be significantly increased yet was undetectable in normal-appearing colonic mucosa, distant from tumours [14].

One study involving samples from breast cancer patients was able to show cJun at particularly high levels at the invasive front of breast cancer tumours compared to benign breast cells [12]. These high levels of cJun were linked with proliferation and angiogenesis and a correlation was found between cJun expression during cell cycle progression and lower survival rate. cJun has also been shown to be strongly overexpressed in cells throughout the tumours of Hodgkin lymphoma patients [17]. A study of acute myeloid leukaemia patient samples showed cJun expression was raised compared to normal bone marrow mononuclear cells [19]. Expression levels were linked to the grade of malignancy in glial tumours, with the role of cJun in producing malignant tumour properties (proliferation, migration and invasion) also illustrated in the same study [16].

The role of cJun in cancer has also been probed using an *ex-vivo* lung cancer model, which indicated that cJun was elevated in circulatory tumour cells compared to primary tumours and metastatic lesions [97]. Overexpression of cJun in MCF-7 cells (a breast cancer cell line) has been shown to induce an invasive cancer phenotype which is clearly linked to the high levels of cJun seen in the invasive edge of tumours from patients [13]. This model system produced a highly relevant feature observed in the clinic, that of hormone resistance. This follows from previous work that showed cJun and cFos inhibit estrogen receptor transcription in MCF-7 cells [98]. This reduction in estrogen receptor protein reduces the inhibitory effect of the drug tamoxifen which functions by binding at these sites. cJun overexpression in these cells is therefore a close mimic of the condition in patients which have been treated with tamoxifen but recur with drug-resistant, aggressive tumours. A further link between cJun/cFos and drug resistance was observed in human leukaemia cells where a drug resistant line was shown to have higher cJun/cFos levels than a drug sensitive line [99].

The involvement of cJun in cancer can also be illustrated by observing the effect of cJun depletion/KO or inactivation within cancer cell lines. In one such piece of work cJun was depleted in Friend murine erythroleukaemia cells by the use of sequence specific antisense oligonucleotides [100]. This was shown to halt proliferation of logarithmically growing cells, pushing them into a resting phase until cJun was restored. In a nasopharyngeal carcinoma cell line, silencing of cJun was shown to decrease cell migration and invasion [101]. Conditional KO of cJun, through a floxed allele, produced significantly fewer tumours in a chemical-induced liver cancer model; this was shown to operate in part through a reduction in cJun antagonism of the proapoptotic protein p53 [102]. Expression of a cJun mutant lacking its TA domain can supress the oncogenic transformation induced by an activated Ras gene in the presence of TPA [103, 104]. Another cJun TA deletion mutant was shown to prevent tumour formation in two malignant mouse epidermal cell lines [105].

### Hijacking Cell Signalling

cJun is active in response to a web of signalling pathways, whereby the inputs of these various signals are integrated into the output of transcriptional activity mediated by cJun binding to TRE (or related) DNA (Fig 2). These signalling pathways are a source of tumorigenic effect on cJun activity, by the production of increased levels of activated cJun. This can be illustrated by the KO of cJun N-terminal phosphorylation in a mouse model of intestinal cancer which reduced tumour number and size and prolonged lifespan [106]. Typically, cJun expression levels remain low until a stimulus elevates levels of activated protein [14, 18]. However, in some cancers these inductive pathways become constitutively activated. For example, this switch to constant activation of cJun (alongside other AP-1 family members and early transcription factors) was shown in human head and neck squamous cell carcinoma cell lines [107]. Cancer can therefore be thought of as hijacking these cellular processes, turning them from responsive pathways to produce conditional activity into permanent signals for growth and migration.

Pathogen sensing, via Toll-like receptor (TLR) proteins, is one such pathway hijacked by cancer (Fig 2). The observed link between prostatitis and prostate cancer [108] has been suggested to occur due to gram-negative bacterial or DNA virus infection in which pathogen LPS bind to TLR proteins [109]. TLR4 expression has been associated with poor progression-free survival in prostate cancer [110], a trend also observed in hepatocellular carcinoma [111]. Subsequent work used silencing of MKK4 or inhibition of JNK activity to show that Toll receptor signalling enhanced hepatocellular carcinoma invasiveness [112]. Taken together this links overactivation of the Toll receptor/JNK pathway with constitutive activation of cJun in cancer. Constitutive activation of JNK has also been shown in glioma cells [113]. The level of activation was correlated with the histological grade of the tumour and EGFR expression. In this case the cancerous cell has hijacked the cells ability to respond to EGF. This JNK activation is crucial for the ability of the glioma cells to remain “stem-like” by self-renewing, and also plays a role in drug resistance [114].

cJun activity is mediated by its ability to persist within cells so alterations in degradation signalling may produce cancer phenotypes. Serine/threonine kinase receptor-associated protein has been shown to be overexpressed in a number of human cancers and is relevant here for its role in cJun regulation [6, 115]. It has been shown to inhibit cJun ubiquitination and therefore proteasomal degradation. This work illustrated how the increased stability of cJun led to increased cyclin D1 expression and increased proliferation. In melanoma, mutant BRAF or N-RAS has been shown to upregulate MAPK signalling [116–118]. It has been shown that the subsequent upregulated activity of ERK produces an increase in cJun transcription and subsequent protein stability [119]. cJun can also be protected from degradation by bZIP binding partners. This was shown to be the case for Fra-1 where RAS-induced overexpression of this oncogenic protein was shown to increase cJun stability [120].

## cJun Antagonism

The literature paints a broad picture of cJun dysregulation across a number of cancers and collectively shows that cJun is generally overactive and as such could be antagonised for therapeutic effect. Cancer related effects can be induced at any level of signalling, but as dysregulation is modulated at the transcriptional endpoint of cJun, antagonism there will ablate any oncogenic signalling in the pathway. Signalling pathways may also modulate multiple TFs and other processes, so attempts to therapeutically interact earlier in the pathway will increase the likelihood of nonspecific effects, which can be undesirable. We generally observe these issues when observing the range of natural products which have been investigated for chemoprotective or chemotherapeutic effects related to AP-1 dysregulation, such as resveratrol and harmal extract [121–123]. They have the potential to produce beneficial outcomes but their mechanism is typically poorly defined; usually due to their action on multiple targets.

**Fig. 3:**
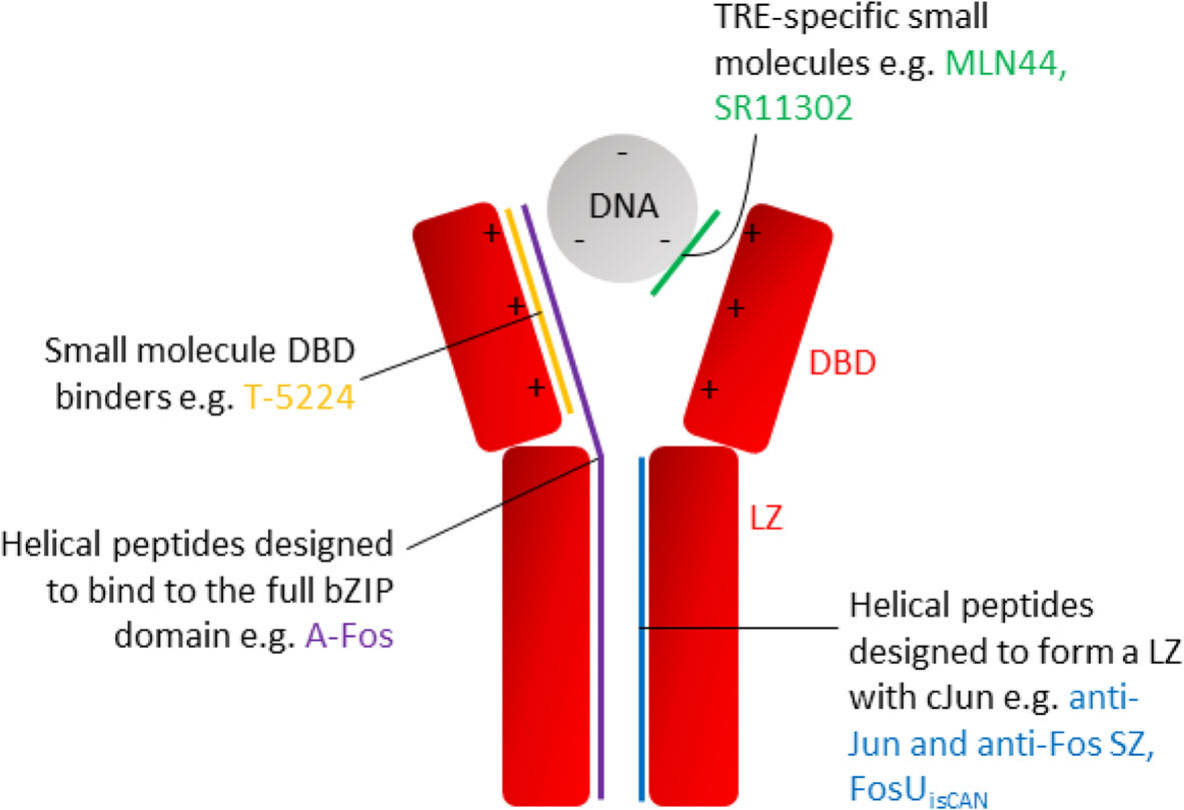
Schematic of cJun binding interfaces and molecules that target them. The cJun-DNA interaction can be antagonised by binding to the TRE site on DNA (MLN44 [129], SR11302 [135]), the cJun DBD (T-5224) [51], the cJun LZ (anti-Jun and anti-Fos SZ [144], FosU_isCan_ [125]) or the full cJun bZIP domain (A-Fos [148])

Although the focus here is cJun it is important to reiterate that some AP-1 components can produce oncogenic effects, and others are anti-oncogenic depending on cellular context and specific dimer composition. A therapeutic would seek to antagonise cJun oncogenic activity selectively while avoiding interaction with closely related AP-1 family members which are functioning normally or may be producing anti-oncogenic effects. Antagonism of cJun can ultimately be achieved by either preventing the bZIP domain from engaging with the DNA or by preventing the binding of the TA domain to any relevant interaction partners. Currently, the most attractive site for cJun antagonism is to directly modulate DNA binding at the bZIP (either by inhibition of dimerisation or by preventing the bZIP from docking to the DNA), since this has already received significant study, whereas little is known about binding at the TA domains of AP-1 proteins. To antagonise complex formation, it is important to study the binding surfaces which bring the functional structure together (Fig 3). This consists of an extended three component interface involving dimerisation of the LZ domains and the interaction of the DBD from each monomer with the DNA. In this section we shall describe a range of cJun antagonists (Table 2), categorised by the binding interface they target, to sketch a picture of the field.

**Table 2:** Summary of known antagonists of the cJun-TRE DNA interaction. Included are known mode of binding as well as quantitative measures of activity for each antagonist identified

Antagonist	Target binding surface	Affinity/Antagonist Activity	Notes	References
MLN44	TRE DNA major groove	100% inhibition in EMSA assay at 25 μM		[129–133]
SR11302	TRE DNA	Treatment prior to TPA induction of tumours produced a 67.9% reduction in papillomas per mouse		[134–136]
Veratramine	TRE DNA	90% reduction in transactivation at 20 μM in luciferase reporter assay	ITC data collected but no binding affinity reported	[137]
KCR motif peptide-1-[N-[2-succinamidylethyl]amino] anthraquinones	TRE DNA	Approaching 100% inhibition in EMSA assay at 1 μM		[138]
T-5224	DBD of AP-1 proteins	IC_50_~10 μM		[51, 139, 140]
NY2267	cJun LZ	74% reduction in transactivation at 20 μM in luciferase reporter assay	Designed as c-Myc antagonist so not selective	[141]
cFos LZ	cJun LZ	For cFos LZ-cJun LZ: K_d_ = 26.6 μM (by ITC)		[142, 143]
JunB bZIP	cJun LZ	Eightfold excess of JunB reduced transactivation tenfold in a luciferase reporter assay		[88]
anti-Jun and anti-Fos SZ	cJun LZ	50% of Jun LZ or Fos LZ bound to the antagonist when the three are mixed in equimolar amounts		[144]
FosW	cJun LZ	For FosW-cJun LZ: K_d_ = 39 nM (by ITC)		[145, 146]
FosW_CANDI_	cJun LZ	For FosW_CANDI_ -cJun LZ: T_m_ = 52 degrees C(by CD)	Reduced affinity with no increase in selectivity compared to FosW	[154]
CPW	cJun LZ	For CPW-cJun LZ: K_d_ = 750 nM (by ITC)		[147]
FosU_isCan_	cJun LZ	For FosU_isCAN_-cJun LZ T_m_ of 57 °C (by CD)		[125]
A-Fos	cJun bZIP	For A-Fos-cJun bZIP: K_d_ = 30 pM (by CD thermal shift from T_m_ of 72.1 °C)		[148]

### Antagonising TRE sites

Drug candidates targeting AP-1 generally and cJun specifically have historically tended to be small molecules. The majority have focused on the DNA TRE site since this presents a small, defined target surface suited to small molecules. MLN44 (or XR5944) is a sequence specific DNA intercalator which has been shown to block cJun binding to DNA containing the TRE site in a dose-dependent manner [129]. Electrophoretic mobility shift assay (EMSA) experiments in this study showed that 25 μM of the compound was required to approach 100% inhibition. Structural NMR studies were used to show how the drug interaction with the DNA major groove precludes cJun DBD insertion. MLN44 was also shown to inhibit transcription and *ex vivo* studies indicated efficacy in a range of cancer tissues [130, 131]. However, an issue of selectivity is common among small molecule DNA binders. As with the TFs they are designed to inhibit, the specificity described for them remains relative, with off target binding possible at related DNA sequences and even non-related sequences. MLN44, for example, has also been shown to bind to the estrogen response element; raising the question of off-target effects [132, 133]. Clinical trials for this molecule appear to have stalled at an early stage.

Retinoids are a vitamin A-related class of molecules, some of which have been shown to bind to TRE DNA sites and are being investigated for their anticancer effects [134]. One TRE-specific retinoid, SR11302, has been investigated in an *ex vivo* lung cancer model and was shown to reduce formation of metastatic lesions [135]. Retinoids unsurprisingly bind to the retinoic acid response element (RARE) in addition to the TRE. It has been shown that the purported antitumour effect of these molecules is mediated by TRE binding and not RARE binding [136]. However, RARE binding does occur and although off target effects like these may not be inherently detrimental, they should be minimised. Veratramine is an alkaloid, derived from *Veratrum* plants, which has also been identified as a selective TRE site binder which can regulate AP-1-dependent gene transcription [137]. This compound at 20 μM was shown to reduce approximately 90% of transactivation activity in a luciferase reporter assay. Further research is required into the selectivity and potential efficacy of this molecule as a cancer therapeutic.

One interesting piece of work went beyond the use of small molecules and incorporated a peptide conjugate. This saw an anthraquinone derivative linked to a small peptide corresponding to a truncated region of the cJun DBD [138]. A conserved motif within all AP-1 protein DBD sequences was utilised that centred around the Lys-Cys-Arg residues (residues 268-270 in cJun; Fig 1c). A number of sequences were tested involving five to seven residues around this motif. This combination of a high affinity non-specific DNA intercalator with the lower affinity but TRE-site specific peptide was shown to displace AP-1 from binding to TRE DNA. The EMSA experimental set up used in this study showed the best construct to be active in low μM concentrations; achieving 74% inhibition at 16.8 μM.

### Antagonising the cJun DBD

If the problem of TRE selectivity were resolutely solved, it is important to note that another selectivity issue would persist, since these molecules would inhibit binding of all AP-1 family proteins (and any other relevant bZIP) to TRE DNA. Some AP-1 proteins that bind TRE sites may have an anti-oncogenic effect, and it is therefore important to block a specific AP-1 dimer, rather than a whole family to promote the desired outcome. Whilst binding to the TRE site can provide therapeutic results, greater selectivity to a single AP-1 component should provide a higher degree of therapeutic control. An additional issue from producing antagonists which bind selectively to the TRE may arise when considering the ability of cJun to bind to SNVs of TRE or CRE sites [53, 57]. A potential antagonist may not possess the same binding promiscuity amongst these related sites as cJun so there may be relevant transcription sites which are not being blocked. Conversely, blocking only the TRE sites may shift the binding equilibria to increase AP-1 binding at non-TRE sites which could also be detrimental.

Blocking the DBD-DNA interaction with increased selectivity could therefore be better achieved by targeting the DBD of cJun rather than the TRE site on the DNA. One example of a molecule binding to the AP-1 DBD exists in the literature: T-5224 [51]. This small molecule is based on a cyclic peptide which was designed to inhibit the cJun-cFos dimer binding to DNA [149]. The cyclic decapeptide Ac-*c* [Cys-Gly-Gln-Leu-Asp-Leu-Ala-Asp-Gly-Cys]-NH_2_ was produced *de novo* by inspection of the cJun and cFos target DBDs and then subsequent experimental optimisation. Computational and NMR methods indicate that the peptide is bound to both DBDs and an enzyme-linked DNA−protein interaction assay indicated an IC_50_ of 8 μM. T-5224 was computationally designed based on this peptide, and cellular assays in relation to arthritis indicated an IC_50_ of 10 μM. T-5224 represents the small molecule inhibitor of AP-1 which has gone the furthest in clinical trials having made it to a discontinued phase II trial for its effectiveness in arthritis. It has also been shown to have anticancer activity in various models [139, 140]. Further clinical study of this molecule (or the peptide from which it is derived) as an anticancer agent may prove fruitful in the future.

### Antagonising the LZ Interface

Sequence alignment of AP-1 proteins (Fig 1c) indicates a high degree of similarity in the DBDs, particularly within the sub-families. This selectivity issue alongside the relatively weak binding affinity of the only known binder to date implies that targeting the cJun DBD will be highly challenging. The LZ domain of cJun exhibits more significant sequence diversity than the DBD so the focus may switch there. Targeting the LZ binding interface raises different challenges which must now be considered. Due to the large surface area of the LZ, which lacks defined binding pockets, small molecules may not bind as effectively. Some small molecules have been developed which bind to the cJun LZ however they were initially produced as c-Myc antagonists, indicating an issue with selectivity [141]. To our knowledge, no specific small molecule cJun LZ binders have yet been developed.

To target the LZ binding surface with significant potential for selective binding, work on protein-based antagonists has instead developed; a less commonly utilised but growing field of study [150, 151]. Peptides can move beyond the Lipinski rules of small molecule drugs and utilise a larger binding surface, which can be particularly useful for protein surfaces without defined binding pockets suited to small molecules. This can produce higher affinity binding than possible with small molecules, which generally operate at lower potency micromolar affinities. Larger surfaces also provide the potential for more target-specific peptide drugs which can reduce toxicity. Peptide-based drugs tend to be less immunogenic because they are composed of natural components that are degraded into safe metabolites (amino acids), which prevents accumulation in tissues. The simplest approach to producing peptides which bind to the cJun LZ is to look to the range of known natural bZIP proteins which bind to cJun. Clearly, the LZ from any bZIP protein which is known to bind to cJun may operate as an antagonist. The cFos-cJun LZ domains in isolation have been shown to bind with a K_d_ of 27 μM by ITC [142]. The potential for antagonism has been shown using cFos and cJun LZ peptides *in vivo* where they inhibited maturation of *Xenopus* oocytes through cJun antagonism [143]. JunB has been shown to bind to the cJun LZ and reduce transactivation according to a luciferase reporter assay [88].

Using WT protein LZ sequences as a guide, one can work towards producing an enhanced antagonist through rational design. LZ design rules to aid in binding selectivity have been studied in the specific case of cJun and related bZIP proteins [152, 153]. By considering the core packing at the **a** and **d** heptad positions and electrostatic interactions at the **e** and **g** positions of the target, selective and high affinity binding of an antagonist can be achieved. Rational optimisation of a WT sequence was performed in one case by Bains *et al* through modification of the cJun LZ [144]. This rationally designed cJun LZ Ala298Val peptide was referred to as an anti-Jun and anti-Fos superzipper (SZ). This simple point mutation at an **a** heptad position in the cJun LZ was predicted to produce more extensive van der Waals interactions with its binding partners. The SZ was subsequently shown to bind to both the cJun and cFos LZ peptides, with a small preference for cFos, using analytical HPLC experiments.

However, maintaining a high level of sequence similarity with native bZIP proteins is likely to present a problem with selectivity as these proteins are known to interact with multiple partners. Optimisation of selective binding to cJun may instead be achieved by the exploration of novel sequence design space. High throughput library screening approaches are being utilised to test large numbers of peptide sequences for cJun LZ binding, allowing for more randomised design. Using an intracellular protein-fragment complementation assay (PCA), a ~62,000 member library was screened to produce a peptide named FosW [145]. This peptide was shown to bind to the cJun LZ with a K_d_ of 39 nM by ITC which illustrates the large increase in binding affinity made possible by targeting this large protein surface [146]. However, FosW was also shown to bind tightly to itself and to cFos (and likely most AP-1 family members).

**Fig. 4:**
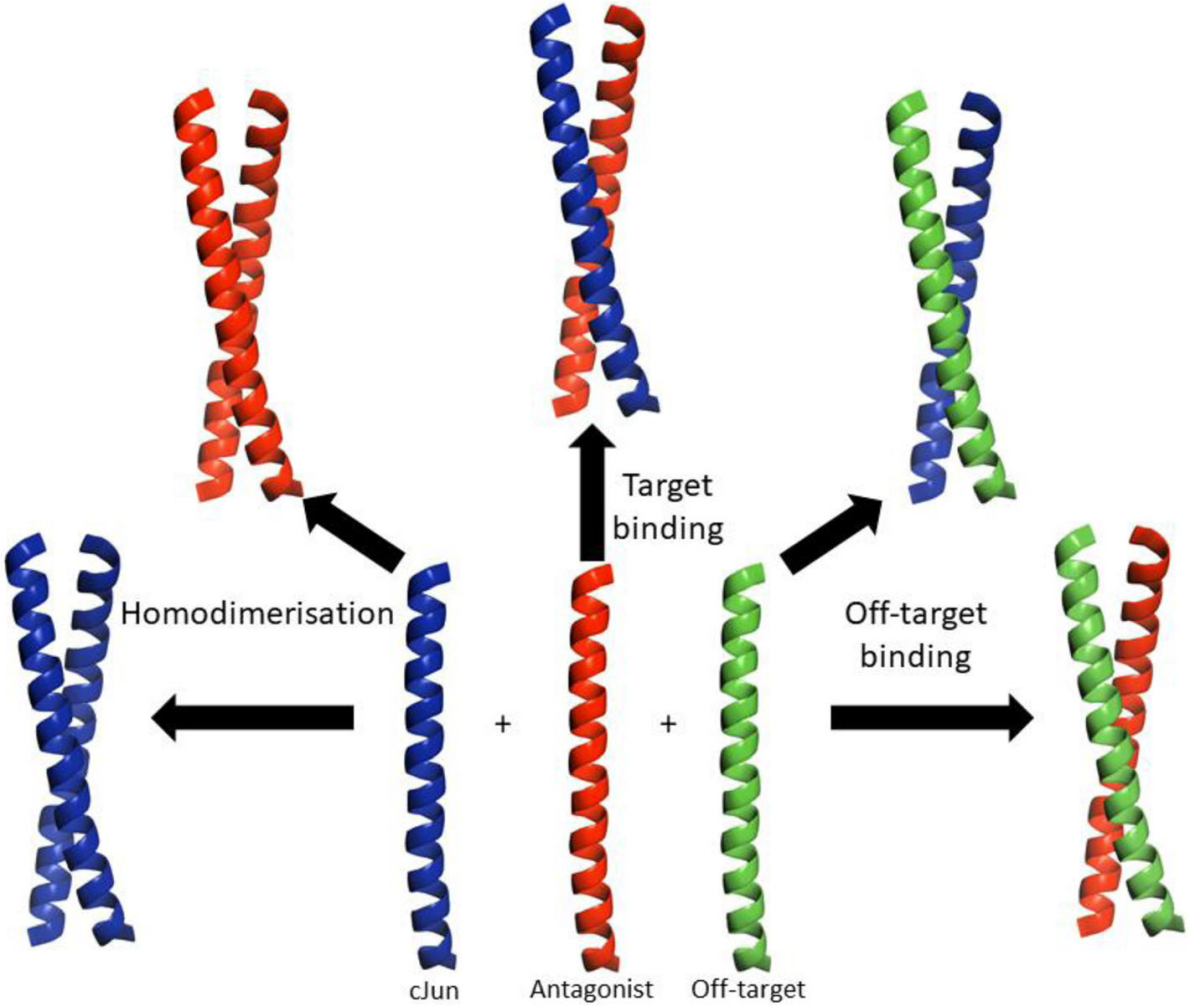
Overview of the potential interactions of a cJun antagonist. This highlights some potential competitive interactions which the antagonist must overcome in order to selectively bind to the cJun target; outcompeting both homodimerisation and interactions with off target components

Clearly a selective cJun antagonist must outcompete the range of possible interactions available in the cellular context (Fig 4). Both target and antagonist can homodimerize and they can also potentially bind to off-target bZIP proteins, so the cJun-antagonist interaction must be preferred over all of these options. The setup for PCA optimises selective target binding over homodimerisation but off-targets are not considered. The Competitive And Negative Design Initiative (CANDI) is an extended version of the PCA assay where off-target proteins are also present; this means selected winner peptides must bind to the target with a greater affinity than the off-target (and other undesirable interactions). The utility of the technique was shown by the generation of a novel peptide that bound specifically to cFos in the presence of cJun, though the attempt to generate a cJun targeting peptide (FosW_CANDI_) did not produce the desired selectivity [154]. Screening of a different peptide library using CANDI methodology may produce better results. Another library screening methodology called CIS display has also been utilised in tandem with PCA to allow for larger libraries to be screened in vitro before further in cell optimisation by PCA to produce a peptide named CPW. This has been shown to bind to the cJun LZ with a K_d_ of 750 nM [147]. A wide range of peptide library screening techniques exists beyond CIS and PCA but these have not yet been used to screen for cJun antagonists.

The exploration of novel sequence space for cJun antagonists can be expanded by the use of computational approaches. There is a prominent research drive to utilise the information encoded in bZIP sequences to computationally predict interactions [124–126, 155, 156]. This work involves the production of large experimental datasets through which predictive computational methods can be developed. This allows the screening of significantly larger libraries of peptide sequences with the caveat that comes with *in silico* approaches: outputs are predictions which must be experimentally tested. Our group used an initial *in silico* screening of a large library to produce a list of antagonist sequences that are predictively ranked by target binding and/or selectivity [125]. This information is then used to produce smaller, higher quality libraries that are accessible to experimental approaches. The PCA screen of this refined library produced FosU_isCan_, which binds tightly to the cJun LZ. Although other peptides are known which bind with higher affinity to the target (such as FosW or CPW), this peptide is significantly more selective due to the lower affinity of homodimerisation and affinity for cFos.

### Antagonising the Full bZIP Domain

To optimise cJun antagonism, it may be pertinent to consider how the components of the target complex exist in equilibrium within cells. The binding of cJun to DNA can potentially occur via two mechanisms. A cJun monomer either binds to the DNA followed by dimerisation with another bZIP or cJun finds a bZIP partner before binding to the DNA as a preformed dimer. Multiple lines of inquiry have indicated that the former is generally preferential for bZIP domains, though the latter also occurs [127, 128, 157]. In the case of the related GCN4 protein, from yeast, the two mechanisms of DNA binding appear to occur at the same rate [158]. It is therefore important to consider both a cJun monomer bound to DNA and a free cJun monomer as our targets for a potential therapeutic.

This raises a potential problem with the antagonists previously discussed; in terms of their functional activity. Whilst an antagonist is bound to the DBD of cJun or the TRE site, dimerisation of cJun may still occur which prepares the protein for DNA binding whenever these antagonists disassociate. Alternatively, an antagonist may be bound to the LZ of cJun whilst the DBD is searching the DNA for a TRE site, ready to form a dimer and influence transcription upon LZ antagonist dissociation. As monomer binding to the DNA is the energetically preferred initiation step, blocking this DBD-DNA interaction is the priority. However, binding to the full bZIP domain inhibits either possible initiation step, and may therefore be considered the preferred route for antagonism. Binding to the full cJun bZIP domain may also allow higher affinity antagonists to be developed due to the larger binding surface available.

The Vinson group has developed a methodology which utilises known LZ antagonists and appends an extension capable of binding to the target DBD [148, 159]. Initially, they rationally designed an acidic extension to bind to the DBD of C/EBP-alpha which they appended to a peptide which binds to the LZ. The rational design involved the incorporation of negatively charged Glu residues to promote intramolecular electrostatic interactions with Arg/Lys sidechains in the DBD. Secondly, the LZ heptad pattern of Leu at the **d** positions was extended into this acidic region in an attempt to extend the LZ packing into the DBD. This rational design was then modified slightly to target the cJun DBD sequence specifically and was appended to the cFos LZ to produce a peptide, referred to as A-Fos. Whether the new acidic domain produces the predicted extended LZ has not been determined, however it has been shown to produce the desired effect by increasing binding affinity for cJun compared to WT cFos. A-Fos was shown to inhibit AP-1 transactivation in a human hepatoma cell line and has subsequently been utilised in other cell based assays where its expression was shown to effectively antagonise cJun-DNA binding [160]. This acidic extension methodology has also been applied by the Vinson group to target CREB and Myc/Max, and by the Keating group to target BZLF1 [161–163].

Targeting the full length of the bZIP domain of cJun is therefore a promising avenue of research to produce powerful functional antagonists which overcome some issues with targeting only the DBD or LZ individually. The potential selectivity issue of targeting the DBD returns here as an antagonist which binds to the DBD of cJun will have at least some relevant degree of affinity for the other AP-1 proteins due to the high sequence homology of their DBDs. This should not prohibit this type of cJun antagonist but must be thoroughly considered nonetheless.

### General Challenges in Antagonising cJun

Despite this range of research, no inhibitor of any AP-1 family member has been approved for clinical use for any disease. Clearly there are some hurdles which must still be overcome. Firstly, we have discussed evidence of both apoptotic or anti-apoptotic activity of cJun depending on cell type [68], and the cJun-dependent upregulation of apoptotic genes such as BIM [96]. These examples highlight that whilst cJun is generally oncogenic, that is not always the case. This may mean that particular tumours are not suited to treatment through cJun antagonism, or that due to the shifting genetic heterogeneity of tumours cJun is only partially effective or loses efficacy over time, but these are common pitfalls of cancer treatments and are therefore not prohibitive to clinical use.

One issue we have not yet raised is the potential for side effects caused by the role of cJun in healthy tissues for the vital response to cellular signals. It may be that antagonism of cJun, which is vital to the functioning of normal processes, may do more harm than the potential cancer therapy it provides. Some studies have noted that cJun can be unobservable in normal tissue but highly overexpressed in tumour tissue [14, 18], which raises the possibility of selectively targeting cancerous cells by cJun antagonism. It may also be possible to modulate the target binding affinity to reach an equilibrium value where an appropriate level of cJun remains unbound. Some degree of encouragement may also be gleaned from the development of Omomyc to target c-Myc, another oncogenic TF [164, 165]. This miniprotein functions, at least in part, by directly antagonising c-Myc-DNA binding. It has shown its therapeutic potential in a range of cancer models. Omomyc and variants are expected to enter clinical trials in 2021 [166, 167]. Whether targeting an essential TF will provide more benefit than side effects is still an open question but in order to test it we must first develop the cJun-specific antagonist tools.

Whilst the use of peptide therapeutics is expanding, there are still significant challenges required to overcome their known issues such as high production cost, bioavailability, biostability and immunogenicity. This may be particularly exacerbated with the larger peptides required to antagonise the full bZIP domain of cJun. A range of methodologies to alleviate the potential short-comings of peptide therapeutics have been developed including systematic downsizing [168, 169], chemical modifications such as acetylation [170–172], incorporation of non-natural amino acids [173, 174], or cyclisation of the peptide using linkages such as lactam bridges [175–180]. It will also be important to consider that cJun localises to the nucleus [181], so peptides may need additional optimisation to promote cellular and nuclear uptake. This can often be achieved by the incorporation of cell penetrating peptides such as penetratin [182], and nuclear localisation signals [183]. Any peptide antagonist will likely require some combination of these modifications to allow development into a successful clinical therapeutic.

## Conclusion

AP-1 serves as a transcriptional super controller, transactivating target genes to modulate a variety of cell signalling pathways. These signals, from an array of sources, alter transcription to control processes such as differentiation, migration, proliferation and apoptosis. As such, oncogenic alteration of these pathways is also coordinated through AP-1 to produce cancer phenotypes through the promotion of growth signal autonomy, angiogenesis, lack of apoptosis and uncontrolled cell migration. A focus on cJun has highlighted the specific evidence of the role of this AP-1 family member in various cancers. The study of small molecules and peptides have both led to progress in the search for antagonists of this oncogenic cJun activity and provided important research tools to probe and further validate the role of cJun in cancer. There have been developments in a variety of surface targets, whether this is the TRE site of DNA or some stretch of the bZIP domain, though none have reached clinical use. This has particularly highlighted the importance of utilising the sequence diversity of the cJun LZ to specifically antagonise cJun oncogenic effect, to avoid antagonism of the potentially anti-oncogenic effect of other AP-1 family members. Functional antagonism, rather than non-functional cJun binding, may be best achieved by an antagonist which binds to the full bZIP domain to prevent both dimerisation and DNA binding simultaneously. With a range of challenges still to be overcome, any method to antagonise cJun-DNA binding may yet prove to be the route to a clinical therapeutic.

## Data Availability

Not applicable.

## Abbreviations

AP-1: Activator protein-1
ATF2: Activating transcription factor 2
BIM: BCL-2 interacting mediator of cell death
bZIP: Basic leucine-zipper
CANDI: Competitive And Negative Design Initiative
CRE: cAMP response element
EMSA: Electrophoretic mobility shift assay
JNK: cJun N-terminal kinase
KO: Knockout
LPS: Lipopolysaccharide
LZ: Leucine zipper
MAPK: Mitogen-activated protein kinase
MEF2C: Myocyte-specific enhancer factor 2C
PCA: Protein-fragment Complementation Assay
RARE: Retinoic acid response element
SNV: Single nucleotide variant
SZ: Superzipper
TA: Transactivation
TF: Transcription factor
TLR: Toll-like receptor
TPA: 12-O-tetradecanoylphorbol-13-acetate
TR: Transrepression
TRE: 12-O-tetradecanoylphorbol-13-acetate response element
